# Enhanced monocyte chemoattractant protein-1 production in aging mice exaggerates cardiac depression during endotoxemia

**DOI:** 10.1186/s13054-014-0527-8

**Published:** 2014-09-11

**Authors:** Hanan Slimani, Yufeng Zhai, Nasser G Yousif, Lihua Ao, Qingchun Zeng, David A Fullerton, Xianzhong Meng

**Affiliations:** Department of Surgery, University of Colorado Denver, Box C-320, 12700 E 19th Avenue, Aurora, CO 80045 USA

## Abstract

**Introduction:**

Endotoxemia and the systemic inflammatory response syndrome have a significant impact on post-surgery outcome, particularly in the elderly. The cytokine response to endotoxin is altered by aging. We tested the hypothesis that vulnerability to endotoxemic cardiac depression increases with aging due to age-related augmentation of myocardial inflammatory responses.

**Methods:**

Adult (4 to 6 months) and old (20 to 22 months) C57/BL6 mice were treated with endotoxin (0.5 mg/kg, iv). Left ventricle (LV) function was assessed using a microcatheter system. Chemokines and cytokines in plasma and myocardium were analyzed by enzyme-linked immunosorbent assay (ELISA). Mononuclear cells in the myocardium were examined using immunofluorescence staining.

**Results:**

Old mice displayed worse LV function (cardiac output: 3.0 ± 0.2 mL/min versus 4.4 ± 0.3 mL/min in adult mice) following endotoxin treatment. The exaggerated cardiac depression in old mice was associated with higher levels of monocyte chemoattractant protein-1 (MCP-1) and keratinocyte chemoattractant (KC) in plasma and myocardium, greater myocardial accumulation of mononuclear cells, and greater levels of tumor necrosis factor-α (TNF-α), interleukin 1β (IL-1β) and interleukin 6 (IL-6) in plasma and myocardium. Neutralization of MCP-1 resulted in greater reductions in myocardial mononuclear cell accumulation and cytokine production, and greater improvement in LV function in old mice while neutralization of KC had a minimal effect on LV function.

**Conclusion:**

Old mice have enhanced inflammatory responses to endotoxemia that lead to exaggerated cardiac functional depression. MCP-1 promotes myocardial mononuclear cell accumulation and cardiodepressant cytokines production, and plays an important role in the endotoxemic cardiomyopathy in old mice. The findings suggest that special attention is needed to protect the heart in the elderly with endotoxemia.

## Introduction

It is well known that cardiac contractile dysfunction caused by bacterial endotoxin is associated with the production of pro-inflammatory mediators [1]. Toll-like receptor 4 (TLR4) plays a central role in the regulation of endotoxin signaling and endotoxin-induced production of multiple pro-inflammatory mediators [2]. We and others have observed that endotoxin induces cardiac contractile depression through upregulation of myocardial production of pro-inflammatory cytokines, such as TNF-α and IL-1β [3-6].

Trauma and stress associated with major surgery can cause gut bacteria translocation, which leads to endotoxemia and the systemic inflammatory response [7,8]. The number of major surgery performed on the elderly is increasing with the increase in life expectancy. The systemic inflammatory response associated with major surgery has a significant impact on the post-surgery outcome in the geriatric population [9,10]. It has been reported that elderly patients with systemic inflammatory response syndrome have higher incidence of morbidity and mortality than younger patients [11]. Although incidence of systemic inflammatory response syndrome and associated mortality in humans is increasing with age, the mechanism of age-associated vulnerability to this syndrome remains unclear. Understanding of the mechanism that regulates the inflammatory responses in the aging heart is important for peri-surgical care in the elderly.

Endotoxemia depresses cardiac function via upregulation of the expression of cardiodepressant cytokines, including TNF-α, IL-1β and IL-6 [4,5,12]. IL-6 expression is elevated in several tissues of old mice [13]. In addition, aging has been shown to exacerbate the cytokine response to pro-inflammatory insults, including endotoxin, trauma, and ischemia/reperfusion injury [14-18]. Thus, it is likely that aging upregulates the myocardial inflammatory responses to endotoxin and exaggerates endotoxemic cardiac depression.

Mononuclear cells are major sources of tissue pro-inflammatory cytokines [19]. While endotoxin induces mononuclear cell infiltration to the myocardium and other tissues [20], the effect of aging on mononuclear cell accumulation in the myocardium during endotoxemia is unclear. Further, the impacts of myocardial mononuclear cell accumulation and associated cytokine production on cardiac functional performance in the aging heart remain to be determined.

We tested the hypothesis that vulnerability to endotoxemic cardiac depression increases with aging due to age-related augmentation of the systemic and myocardial inflammatory responses. The purposes of this study are: 1) to examine whether aging mice have exaggerated cardiac contractile depression when exposed to endotoxin, 2) to determine whether endotoxemic cardiac depression, as a function of age, correlates with the levels of systemic and myocardial inflammatory responses and 3) to identify the factor that is responsible for the cytokine response and cardiac depression in aging mice.

## Materials and methods

### Animals and treatment

Adult (4 to 6 months) and old (20 to 22 months) male C57BL/6 mice were obtained from the Jackson Laboratory (Bar Harbor, Maine, USA) and National Institute on Aging (Bethesda, MD, USA). Mice were acclimated for 14 days in a 12:12-h light-dark cycle with free access to water and regular chow diet before the experiments. The experiments were approved by the Institutional Animal Care and Use Committee of the University of Colorado Denver, and this investigation conforms to the Guide for the Care and Use of Laboratory Animals (National Research Council, revised 1996).

Adult and old animals were assigned to one of the following experimental groups (n = 6 in each group): normal saline group, lipopolysaccharide (LPS) group, LPS + monocyte chemoattractant protein-1 (MCP-1)-neutralizing antibody group and LPS + keratinocyte chemoattractant (KC)-neutralizing antibody group. All treatments were performed in the morning. LPS (*Escherichia coli* 0111:B4, Sigma Chemical Co, Saint Louis, MO, USA) in a dose of 0.5 mg/kg was injected through a tail vein. Control animals were treated with the same volume of sterile normal saline. Neutralizing antibody against MCP-1 or KC (R&D Systems, Minneapolis, MN, USA) was injected through a tail vein 1 h after injection of LPS. The animals were observed for 6 h after injection of LPS or saline. After analysis of cardiac function, the heart tissue and blood were collected and prepared for analysis of chemokines and cytokines.

### Measurement of cardiac function

We assessed cardiac function at 6 h after LPS treatment as described previously [21,22]. Briefly, mice were anesthetized with pentobarbital sodium (Vortech Pharmaceuticals, Dearborn, MI, USA; 50 mg/kg, intraperitoneal (ip)) and anticoagulated with heparin (Elkins-Sinn, Cherry Hill, NJ, USA; 1,000 units/kg, ip). Animals were laid supine on a heating blanket and core body temperature was maintained at 37°C ± 0.5°C. A microcatheter (Millar Instruments, Houston, TX, USA; 1 F) was inserted into the left ventricle (LV) through the right common carotid artery. Pressure-volume loop was recorded using the MPVS-400 system with the aid of PVAN software (Millar Instruments). Heart rates, LV pressure, LV volume, and related function parameters were analyzed: 10 μL of 30% saline was injected into the inferior vena cava for actual ventricle volume calculation.

### Immunofluorescence staining

The optimal cutting temperature (OCT)-embedded tissues were cut into thin sections (5-μm thick). Sections were treated with a mixture of 30% acetone and 70% methanol for 5 minutes, washed with PBS and fixed with 4% paraformaldehyde. Then sections were incubated with a rabbit polyclonal antibody against murine CD68 (a marker of mononuclear cells, including monocytes and macrophages) followed by Cy3-tagged goat anti-rabbit IgG (imaged on the red channel). Nuclei were stained with bis-benzimide (4′,6-diamidino-2-phenylindole (DAPI), imaged on the blue channel), and glycoproteins on cell surfaces were stained with Alexa 488-tagged wheat germ agglutinin (imaged on the green channel). Microscopy was performed with a Leica DMRXA digital microscope (Leica Mikroskopie and System GmbH, Wetzlar, Germany). Images were analyzed in a blinded fashion.

### ELISA

Commercial ELISA kits (R & D Systems) were utilized to quantify chemokines (MCP-1, KC and macrophage inflammatory protein-1α (MIP-1α)) and cytokines (TNF-α, IL-1β and IL-6) in plasma and myocardial tissue homogenates, as well as cardiac troponin-I (cTn-I) in plasma. Samples and standards were prepared according to manufacturer's instructions. Absorbance of standards and samples were determined spectrophotometrically at 450 nm, using a microplate reader (Bio-Rad Laboratories, Inc, Hercules, CA, USA). Results were plotted against the linear portion of a standard curve.

### Statistical analysis

Data are presented as mean ± standard error (SE). Statistical analysis was performed using StatView software (Abacus Concepts, Calabasas, CA, USA). Analysis of variance (ANOVA) with Fisher post-hoc test was used to analyze differences between experimental groups, and differences were confirmed using the Mann-Whitney *U-*test. Statistical significance was defined as *P* ≤0.05.

## Results

### Endotoxemia results in worse cardiac depression in old mice

**Figure 1:**
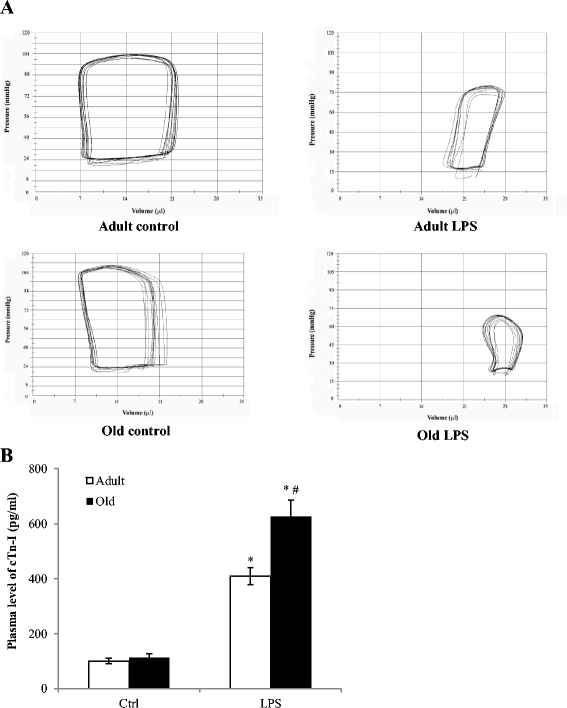
**Endotoxemia results in greater cardiac depression and worse myocardial injury in old mice.** Adult and old mice were treated with lipopolysaccharide (LPS, 0.5 mg/kg, iv) or normal saline. Left ventricular pressure-volume loops and plasma levels cardiac troponin-I (cTn-I) were analyzed 6 h after treatment. Old mice displayed greater reduction in left ventricular performance **(A)** and higher levels of cTn-I in plasma **(B)**. Data are expressed as mean ± standard error; *P <0.05 versus corresponding control (Ctrl); ^#^ *P <*0.05 versus adult mice with LPS treatment.

We determined whether endotoxemia causes greater cardiac depression in old mice than in adult mice. Heart rate increased in both adult and old mice treated with endotoxin in comparison to saline-treated controls (Table 1). Compared to the control group, ejection fraction and developed pressure decreased in endotoxin-treated adults and old mice. Similarly, cardiac output was decreased in both adult and old mice after endotoxin treatment. The representative pressure-volume loops in Figure 1A and analyzed data in Table 1 show significantly greater decreases in LV function in old mice. Thus, endotoxemia causes a reduction in LV function, and old mice have worse LV function during endotoxemia.

**Table 1:** **Left ventricle function parameters**

**Parameter**	**Control**		**LPS**		**LPS + KC NAb**		**LPS + MCP-1 NAb**	
	**Adult**	**Old**	**Adult**	**Old**	**Adult**	**Old**	**Adult**	**Old**
Heart rate (bpm)	430 ± 13	427 ± 15	490 ± 25*	530 ± 23*	481 ± 18*	508 ± 20*	492 ± 30*	501 ± 20*
Developed pressure (mmHg)	83.0 ± 5.0	79.0 ± 3.5	57.0 ± 2.0*	46.0 ± 1.9*^†^	62.0 ± 4.5*	51.1 ± 5.0*^†^	73.1 ± 4.0*^#^	68.2 ± 2.7*^#^
End-systolic volume (μL)	7.0 ± 2.3	8.1 ± 2.5	18.0 ± 2.0*	24.5 ± 2.2*^†^	18.2 ± 1.5*	23.5 ± 1.1*^†^	11.0 ± 1.0*^#^	15.0 ± 1.5*^†#^
End-diastolic volume (μL)	21.0 ± 1.5	20.1 ± 1.7	27.0 ± 1.8*	30.1 ± 2.8*	28.0 ± 1.5*	30.1 ± 3.1*	22.0 ± 1.1^#^	24.0 ± 1.8^#^
Ejection fraction (%)	66.1 ± 4.2	60.0 ± 4.8	33.1 ± 2.8*	20.0 ± 1.8*^†^	37.1 ± 2.5*	22.1 ± 1.8*^†^	49.1 ± 3.7*^#^	39.0 ± 2.6*^†#^
Cardiac output (mL/min)	5.9 ± 0.4	5.2 ± 0.4	4.4 ± 0.3*	3.0 ± 0.2*^†^	4.8 ± 0.3*	3.3 ± 0.3*^†^	5.4 ± 0.3^#^	4.5 ± 0.4^#^

Mice were treated with lipopolysaccharide (LPS, 0.5 mg/kg) for 6 h. Both adult and old mice displayed significantly reduced left ventricle (LV) function. Old mice displayed worse LV performance, including developed pressure, ejection fraction and cardiac output, compared with adult mice. Treatment with monocyte chemoattractant protein-1(MCP-1)-neutralizing antibody (NAb) improved LV function in both adult and old mice, and old mice had a greater improvement. In contrast, neutralization of keratinocyte chemoattractant (KC) had a minimal effect on LV function. Data are expressed as mean ± standard error. ^*^ *P* <0.05 versus corresponding control; ^†^ *P* <0.05 versus adult mice with the same treatment; ^#^ *P* <0.05 versus LPS alone.

To examine whether exaggerated cardiac depression in old mice is associated with worse myocardial injury, we analyzed cardiac Tn-I levels in the plasma, as it is a reliable marker of myocardial injury [23]. As shown in Figure 1B, treatment with LPS caused a significant increase in plasma levels of cardiac Tn-I in both adult and old mice, although the concentrations were not very high. The release of cardiac Tn-I was greater in old mice (626 ± 40 pg/mL in old mice versus 410 ± 25 pg/mL in adult mice; *P* <0.05, n = 6). Therefore, the exaggerated LV dysfunction in old mice is associated with augmented myocardial injury.

### Aging augments MCP-1 and KC production during endotoxemia

**Figure 2:**
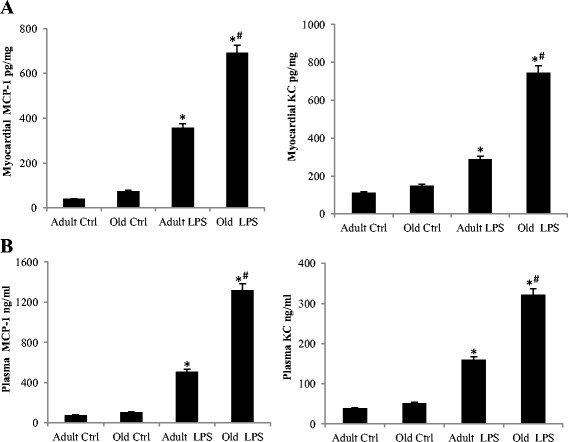
**Old mice have higher levels of chemokines in the myocardium and plasma.** Adult and old mice were treated with lipopolysaccharide (LPS, 0.5 mg/kg, iv) or normal saline. Levels of MCP-1 and KC were analyzed by ELISA 6 h after treatment. Old mice had higher levels of MCP-1 and KC in myocardial tissue **(A)** and plasma **(B)**. Data are expressed as mean ± standard error. *P <0.05 versus corresponding control (Ctrl); ^#^ *P <*0.05 versus adult mice treated with LPS.

Treatment with endotoxin increased the levels of MCP-1 and KC in plasma and the myocardium (Figure 2A and B). Interestingly, old mice had significantly higher levels of MCP-1 and KC in plasma and the myocardium than adult mice. While MIP-1α levels also increased after endotoxin treatment, there was no significant difference in MIP-1α levels between adult and old mice (data not shown).

### Old mice display greater myocardial mononuclear cell accumulation and cytokine production

**Figure 3:**
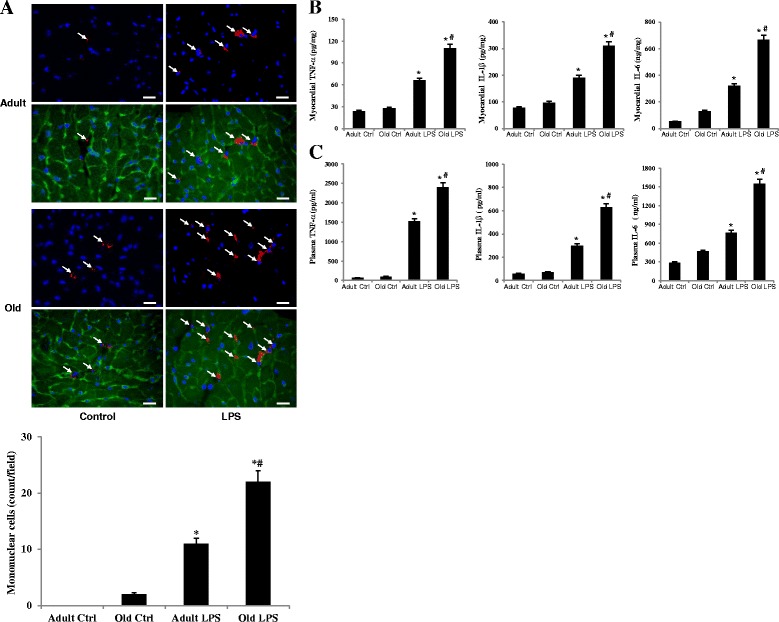
**Myocardial mononuclear cell accumulation and pro-inflammatory cytokine production are enhanced in old mice.** Adult and old mice were treated with lipopolysaccharide (LPS, 0.5 mg/kg, iv) or normal saline. Mononuclear cells in the myocardium were detected by immunofluorescence staining using a polyclonal antibody against murine CD68 (red), and levels of TNF-α, IL-1β, and IL-6 in the myocardium and plasma were measured by ELISA 6 h after LPS treatment. The myocardium of old mice displayed higher densities of mononuclear cells (arrow) after LPS treatment (**A)** (size bar = 20 μm). In addition, the levels of TNF-α, IL-1β, and IL-6 were higher in the myocardium **(B)** and plasma **(C)** of old mice compared to adult mice. Data are expressed as mean ± standard error. *P <0.05 versus corresponding control (Ctrl); ^#^ *P <*0.05 versus adult mice treated with LPS.

We analyzed mononuclear cell accumulation in myocardial tissue, as well as the levels of pro-inflammatory cytokines (TNF-α, IL-1β and IL-6) in plasma and myocardial tissue of adult and old mice. The number of mononuclear cells increased following endotoxin treatment (Figure 3A). Old mice, however, exhibited greater mononuclear cell accumulation in the myocardium (Figure 3A).

As shown in Figure 3B, levels of TNF-α, IL-1β and IL-6 increased in myocardial tissue after endotoxin treatment in adult and old mice. Old mice, however, displayed higher levels of myocardial TNF-α (110 ± 10 pg/mL in the old versus 66 ± 5 pg/mL in adults; *P* <0.05, n = 6), IL-1β (310 ± 25 pg/mL in the old versus 190 ± 13 pg/mL in adult; *P* <0.05, n = 6) and IL-6 (667 ± 51 ng/mL in the old versus 322 ± 17 ng/mL in adult; *P* <0.05, n = 6) compared to adult mice. Similar differences between adult and old mice were observed in plasma levels of these cytokines (Figure 3C). Thus, augmented myocardial production of cardiodepressant cytokines in aging mice is associated with enhanced mononuclear cell accumulation in the myocardium.

### Neutralization of MCP-1, not KC, in old mice reduces myocardial mononuclear cell accumulation and cytokine production

**Figure 4:**
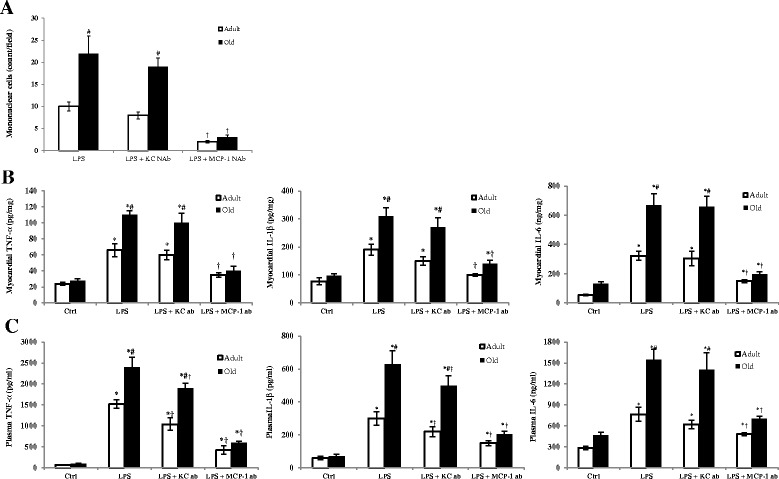
**Neutralization of monocyte chemoattractant protein-1 (MCP-1) reduces myocardial mononuclear cell accumulation and cytokine production in old mice.** MCP-1-neutralizing antibody or keratinocyte chemoattractant (KC)-neutralizing antibody (NAb or ab; 8 μg/mouse, iv) were administered 60 minutes after injection of lipopolysaccharide (LPS, 0.5 mg/kg, iv). Mononuclear cells in the myocardium were detected by immunofluorescence staining, and levels of TNF-α, IL-1β, and IL-6 in the myocardium and plasma were analyzed by ELISA 6 h after LPS treatment. Neutralization of MCP-1, not KC, reduced myocardial mononuclear cell density **(A)** and cytokine levels in the myocardium and plasma **(B and C)**. Greater reductions were observed in old mice. Data are expressed as mean ± standard error. *P <0.05 versus corresponding control (Ctrl); ^#^ *P <*0.05 versus adult mice treated with LPS alone or LPS + KC-NAb; †*P <*0.05 versus corresponding age group treated with LPS alone or LPS + KC-Nab.

We applied specific neutralizing antibodies to determine the role of MCP-1 and KC in myocardial mononuclear cell accumulation and cytokine production in adult and old mice. In comparison to mice treated with endotoxin alone, mice treated with endotoxin plus MCP-1-neutralizing antibody had markedly lower densities of mononuclear cells in the myocardium (Figure 4A). While the reduction in mononuclear cell accumulation was observed in both adult and old mice, a greater reduction was attained in old mice, resulting in the abrogation of an age-related difference. In contrast, neutralization of KC had a minimal effect on myocardial mononuclear cell accumulation in either adult or old mice (Figure 4A).

Myocardial cytokines (TNF-α, IL-1β and IL-6) levels were also markedly reduced in mice treated with endotoxin plus MCP-1-neutralizing antibody in comparison to mice treated with endotoxin alone (Figure 4B). However, neutralizing KC had a minimal effect on myocardial production of these cytokines although plasma levels of TNF-α and IL-1β are moderately reduced by KC-neutralizing antibody (Figure 4C). Again, the effect of MCP-1 neutralization is more robust in aging mice, and the age-related differences in myocardial cytokine levels are essentially abolished (Figure 4B). Similar results for plasma cytokine levels were obtained (Figure 4C). Thus, neutralization of MCP-1 reduced mononuclear cell accumulation in the myocardium and suppressed cardiodepressant cytokine production during endotoxemia, particularly in old mice.

### Neutralization of MCP-1 reduces myocardial injury and improves LV function in old endotoxemic mice

**Figure 5:**
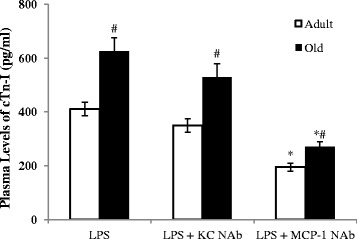
**Neutralization of monocyte chemoattractant protein-1 (MCP-1) reduces myocardial injury.** MCP-1-neutralizing antibody or keratinocyte chemoattractant (KC)-neutralizing antibody (NAb; 8 μg/mouse, iv) were administered 60 minutes after injection of lipopolysaccharide (LPS, 0.5 mg/kg, iv). Plasma levels of cardiac troponin-I (cTn-I) were analyzed by ELISA 6 h after LPS treatment. Neutralization of MCP-1, not KC, reduced cTn-I levels in plasma. Old mice displayed a greater reduction although cTn-I levels remain higher than those of adult mice receiving the same treatment. Data are expressed as mean ± standard error. *P <0.05 versus corresponding age group treated with LPS alone; ^#^ *P <*0.05 versus adult mice receiving the same treatment.

We determined the effect of neutralization of MCP-1 or KC on LV function and myocardial injury in adult and old mice. We found that LV performance was improved by administration of MCP-1-neutralizing antibody in both adult and old mice (Table 1). In comparison to mice treated with endotoxin alone, developed pressure increased by 48% in old mice treated with endotoxin plus MCP-1-neutralizing antibody, and it increased by 28% in adult mice receiving the same treatment. Ejection fraction in old mice treated with endotoxin plus MCP-1-neutralizing antibody increased by 95% while it increased by 48% in adult mice receiving the same treatment. Similarly, cardiac output increased by 50% and 23% in old mice and adult mice, respectively, by treatment with MCP-1-neutralizing antibody. However, KC-neutralizing antibody had a minimal effect on LV function parameters in either adult or old endotoxemic mice. As shown in Figure 5, neutralization of MCP-1 also reduced plasma levels of cardiac Tn-I in both adult and old mice, with a bigger reduction in the latter. Noticeably, a minimal reduction in plasma levels of cardiac Tn-I was achieved by neutralization of KC in either adult or old mice.

## Discussion

In the present study, we observed that old mice exhibit worse LV function during endotoxemia. Exaggerated cardiac depression in old mice is associated with higher levels of MCP-1 and KC in plasma and myocardium, greater myocardial accumulation of mononuclear cells (monocytes and macrophages), augmented production of TNF-α, IL-1β and IL-6 in the myocardium, and worse myocardial injury. Neutralization of MCP-1 results in greater reductions in mononuclear cell accumulation and cytokine production in the myocardium, attenuates myocardial injury and markedly improves LV function, particularly in old mice. Thus, MCP-1 plays an important role in myocardial inflammatory responses and cardiac dysfunction caused by endotoxin in old mice.

### Worse myocardial injury and cardiac depression in old mice are associated with augmented cytokine production

Elderly patients with sepsis or septic shock have a higher mortality rate [24]. During sepsis, the inflammatory responses mediate cardiovascular abnormalities, including vascular endothelial cell damage, increased vascular permeability, hypotension and cardiac contractile dysfunction [25]. To understand whether there is an age-related difference in the vulnerability to endotoxemic cardiac depression, the present study compared LV function in adult and old mice following endotoxin treatment. We observed that LV function is compromised after administration of endotoxin in both adult and old mice. However, old mice display worse LV function in comparison to adult mice when being challenged with the same dose of endotoxin. It is evident that developed pressure and ejection fraction are lower in old endotoxemic mice. Similarly, old endotoxemic mice have lower cardiac output. These results demonstrate that old mice are more vulnerable to endotoxemic cardiac depression, displaying greater reductions in LV contractility and overall cardiac performance. In addition, the greater increase in circulating levels of cardiac Tn-I in old mice indicates greater myocardial injury.

A number of studies have demonstrated that cardiac dysfunction during endotoxemia is caused by inflammatory cytokines, including TNF-α, IL-1β and IL-6 [3-6,12,22]. In addition, pro-inflammatory cytokines have been implicated in cardiac dysfunction after acute injuries caused by burns [26], sepsis [27], and myocardial ischemia and reperfusion [28]. Intravenous administration of either TNF-α or IL-1β can induce a similar change to that caused by endotoxemia and endotoxic shock [29,30], and cardiovascular abnormalities, and mortality can be ameliorated by applying IL-1β receptor antagonist or anti-TNF-α antibodies [31,32]. Previous studies by our group and others demonstrated that intercellular adhesion molecule-1 (ICAM-1) also plays an important role in the endotoxemic cardiac dysfunction and that TNF-α links TLR4 activation to myocardial ICAM-1 expression [6,33]. In the present study, we observed that endotoxin increases the levels of TNF-α, IL-1β and IL-6 in plasma and myocardial tissue of adult and old mice, and the cytokine response is augmented in old mice. While higher levels of TNF-α, IL-1β and IL-6 in plasma and tissues of old endotoxemic animals have been reported [14,16-18], few studies have determined the relationship between the augmented cytokine response and cardiac performance in old animals. The results of the present study show that the worse cardiac performance in old mice is associated with higher levels of TNF-α, IL-1β and IL-6 in plasma and myocardial tissue. Our results suggest that significantly higher levels of cardiodepressant cytokines in the myocardium contribute, at least partly, to the mechanism of exaggerated cardiac depression in old mice.

Previous studies suggest that inducible nitric oxide synthase (iNOS) and nitric oxide (NO) mediate the effect of myocardial depressant cytokines, including TNF-α, IL-1β and IL-6 [3,5,34]. Indeed, NO and peroxynitrite inhibit myocardial O_2_ consumption [35], and reduce the heart’s ability to utilize ATP for contractile work [36]. However, our previous study demonstrates that pro-inflammatory cytokines contribute cardiac depression in this endotoxemia model through an NO-independent mechanism [37]. It is likely that these cytokines directly depress cardiac contractility and induce myocardial injury. In this regard, we have observed that perfusion of TNF-α to isolated hearts results in myocardial injury and contractile depression [28]. In addition, endotoxin and cytokines have been shown to directly depress the contractility in cultured cardiomyocytes [12,38]. It is noteworthy that myocardial injury could contribute to cardiac functional deficit caused by endotoxin although the levels of circulating cardiac Tn-I is rather low, even in old mice exposed to endotoxin.

### Enhanced MCP-1 production plays an important role in upregulating cytokine response and mediating LV dysfunction in old mice

Augmented production of chemokines, that is, KC and MCP-1, in response to endotoxin is observed in both plasma and myocardial tissue of old mice. It is possible that the greater levels of pro-inflammatory cytokines in the myocardium of old mice are due to chemokine-mediated leukocyte accumulation in the myocardium. Neutrophil infiltration occurs in the myocardium during endotoxemia or endotoxic shock [39,40], and IL-8 is an important chemoattractant factor for neutrophils [41]. In the present study, we observed a significant increase in circulating and myocardial levels of KC, a murine analog of human IL-8, in mice treated with endotoxin. However, we previously observed moderate myocardial neutrophil infiltration in this model of endotoxemia induced by a low dose of endotoxin, and depletion of neutrophils with a monoclonal antibody has no effect on cardiac dysfunction in this model [33]. Furthermore, neutralization of KC has no significant effect on LV function. Thus, myocardial neutrophils have not been the central theme of this study.

Infiltrated mononuclear cells are important sources of tissue pro-inflammatory cytokines [42]. MCP-1 is a member of the C-C chemokine family, and a potent chemotactic factor for monocytes [43]. MCP-1 is produced by many cell types and regulates the migration and infiltration of monocytes and lymphocytes. Neutralization of MCP-1 has been shown to reduce leukocyte recruitment and prevent tissue injury in a rat model of endotoxin-induced intestinal muscularis and gastrointestinal dysmotility [44]. In the present study, we observed that MCP-1 levels in plasma and myocardial tissue are significantly higher in the old mice than adult mice after administration of endotoxin, which is accompanied by higher levels of mononuclear cell accumulation in the myocardium and greater production of pro-inflammatory cytokines. MCP-1-neutralizing antibody reduces mononuclear cell accumulation, as well as pro-inflammatory cytokine production, in the myocardium of old mice. In contrast, neutralization of KC has a minimal effect on myocardial mononuclear cell accumulation and cytokine production although it reduces plasma levels of TNF-α and IL-1β. The results of the present study support the notion that MCP-1 plays an important role in the overall myocardial inflammatory responses, and enhanced MCP-1 production is responsible for greater myocardial mononuclear cell accumulation and cytokine production in old mice. However, it should be noted that myocardia cytokines can come from multiple additional sources, including resident macrophages, microvascular endothelial cells, fibroblasts and cardiomyocytes. The effects of MCP-1-neutralizing antibody on mononuclear cell accumulation and myocardial cytokine production only show an association of these two inflammatory events. It remains unclear from this study whether infiltrated mononuclear cells are the main source of myocardial cytokines and whether MCP-1 has any direct or indirect effect on other cells in the heart that are capable of producing cytokines. These questions need to be answered by future studies.

Importantly, we observed that treatment with MCP-1-neutralizing antibody results in a greater improvement in LV function in old mice than in adult mice. For example, ejection fraction was improved by 48% in adult mice, but it was improved by 95% in old mice. While the age-related differences remained in some LV functional parameters, such as ejection fraction, the differences became much smaller following treatment with MCP-1-neutralizing antibody. Thus, neutralization of MCP-1 improved, but did not normalize, LV function in both adult and old mice and reduced the difference in LV functional deficit between adult and old mice. It should be noted that neutralization of KC had a minimal effect on LV function in either adult or old mice although it moderately reduced plasma levels of TNF-α and IL-1β. This finding indicates the importance of myocardial cytokines, rather than circulating cytokines, in LV dysfunction. The beneficial effect of MCP-1 neutralization on cardiac function appears to be through reducing myocardial production of cardiodepressant cytokines and myocardial injury since markedly improved LV function in old mice treated with MCP-1-neutralizing antibody correlates to greater reductions in myocardial levels of TNF-α, IL-1β and IL-6, and in plasma levels of cardiac Tn-I. While both reduced myocardial inflammatory responses and attenuated myocardial injury appear to account for the marked improvement in LV function in old mice, the myocardial injury may have a minor role in cardiac dysfunction in this endotoxemia model. Noticeably, circulating levels of cardiac Tn-I remain higher in old animals when the age-related difference in cardiac output is abrogated.

## Conclusions

Augmented myocardial cytokine production in response to endotoxin results in worse cardiac depression in old mice. Endotoxin induces greater MCP-1 production in old mice, and this chemoattractant protein plays an important role in the augmentation of myocardial mononuclear cell accumulation and cardiodepressant cytokine production in old mice, leading to exaggerated myocardial injury and worse LV functional performance. Thus, MCP-1 appears to be a potential therapeutic target for suppression of myocardial inflammatory responses and for protection of cardiac function in the elderly with endotoxemia.

### Limitations

The endotoximia model has limitations. Although endotoxin is involved in several clinical conditions, such as sepsis and systemic inflammatory response syndrome, it is only one of the multiple factors that mediate tissue inflammatory responses and injury seen in the clinical settings. Investigation of the effect of a single factor, that is, endotoxin, can improve the understanding of the mechanism by which this factor exerts its effect. However, observations made in a simplified model could not be extrapolated to the complicated situation of sepsis and systemic inflammatory response syndrome. In addition, cardiac function was analyzed under anesthesia. The effect of anesthetics may introduce certain variability to the cardiac functional parameters.

## Key messages

1. Old mice display exaggerated cardiac functional depression during endotoxemia that is accompanied by enhanced myocardial inflammatory responses.
2. MCP-1 promotes myocardial mononuclear cell accumulation and cardiodepressant cytokine production, and plays an important role in the endotoxemic cardiomyopathy in old mice.
3. Special attention may be needed to protect the heart in the elderly with endotoxemia.

## Abbreviations

cTn-1: cardiac troponin-I
ELISA: enzyme-linked immunosorbent assay
ICAM-1: intercellular adhesion molecule-1
IL-1β: interleukin 1β
IL-6: interleukin 6
IL-8: Interleukin 8
iNOS: inducible nitric oxide synthase
KC: keratinocyte chemoattractant
LPS: lipopolysaccharide
LV: left ventricle
MCP-1: monocyte chemoattractant protein-1
MIP-1α: macrophage inflammatory protein-1α
NO: nitric oxide
PBS: phosphate-buffered saline
TLR4: toll-like receptor 4
TNF-α: tumor necrosis factor-α
